# Evaluation of the effects of titanium dioxide nanoparticles on cultured *Rana catesbeiana* tailfin tissue

**DOI:** 10.3389/fgene.2013.00251

**Published:** 2013-11-21

**Authors:** S. Austin Hammond, Amanda C. Carew, Caren C. Helbing

**Affiliations:** Department of Biochemistry and Microbiology, University of VictoriaVictoria, BC, Canada

**Keywords:** nanometal, nanoparticle, titanium dioxide, thyroid hormone, amphibian, organ culture, oxidative stress

## Abstract

Nanoparticles (NPs), materials that have one dimension less than 100 nm, are used in manufacturing, health, and food products, and consumer products including cosmetics, clothing, and household appliances. Their utility to industry is derived from their high surface-area-to-volume ratios and physico-chemical properties distinct from their bulk counterparts, but the near-certainty that NPs will be released into the environment raises the possibility that they could present health risks to humans and wildlife. The thyroid hormones (THs), thyroxine, and 3,3′,5-triiodothyronine (T_3_), are involved in development and metabolism in vertebrates including humans and frogs. Many of the processes of anuran metamorphosis are analogous to human post-embryonic development and disruption of TH action can have drastic effects. These shared features make the metamorphosis of anurans an excellent model for screening for endocrine disrupting chemicals (EDCs). We used the cultured tailfin (C-fin) assay to examine the exposure effects of 0.1–10 nM (~8–800 ng/L) of three types of ~20 nm TiO_2_ NPs (P25, M212, M262) and micron-sized TiO_2_ (μ TiO_2_) ±10 nM T_3_. The actual Ti levels were 40.9–64.7% of the nominal value. Real-time quantitative polymerase chain reaction (QPCR) was used to measure the relative amounts of mRNA transcripts encoding TH-responsive THs receptors (*thra* and *thrb*) and *Rana* larval keratin type I (*rlk1*), as well as the cellular stress-responsive heat shock protein 30 kDa (*hsp30*), superoxide dismutase (*sod*), and catalase (*cat*). The levels of the TH-responsive transcripts were largely unaffected by any form of TiO_2_. Some significant effects on stress-related transcripts were observed upon exposure to micron-sized TiO_2_, P25, and M212 while no effect was observed with M262 exposure. Therefore, the risk of adversely affecting amphibian tissue by disrupting TH-signaling or inducing cellular stress is low for these compounds relative to other previously-tested NPs.

## Introduction

Nanoparticles (NPs) are materials that have been purposefully manufactured to the nanometer scale (Handy et al., 2008). They are used in a diverse array of consumer goods and industrial processes, and their use is expected to expand in the future (Oberdörster et al., 2007). This utility derives from the unique properties of materials at this scale: the high surface-area-to-volume ratio is thought to raise the reactivity relative to the bulk form of the material (Wijnhoven et al., 2009). Titanium dioxide NPs are increasingly entering the marketplace due to their usefulness in multiple applications. The estimated current production is approximately 44,400 tonnes (2.5% of total TiO_2_ production), but by 2015 it is expected to reach approximately 260,000 tonnes (10% of total production) (Robichaud et al., 2009). These NPs are used in personal care products such as sunscreens, toothpastes, and cosmetics, industrial products such as paints, lacquers, and papers, and photocatalytic processes such as water treatment (Schmid and Riediker, 2008; Robichaud et al., 2009). The growth of the NP industry indicates an increased likelihood that significant quantities will be released into the environment (Scown et al., 2010). Possible routes of environmental release include production and transport spills, production wastes, and from products during their use, reuse, and end disposal (Baun et al., 2009). Due to these exposure risks, increased attention is being paid to possible negative health effects of NPs on humans, along with the concern that wildlife and the environment may be affected as well (Oberdörster et al., 2007; Handy et al., 2008; Baun et al., 2009).

Although TiO_2_ NPs have been used as non-bioactive controls in nanotoxicology studies, exposure to 1 mg/L P25 TiO_2_ NPs affected 170 gene transcripts in *Danio rerio* after 48 h (Griffitt et al., 2009). Realistically, wildlife are exposed to lower concentrations and, although it is not yet possible to reliably determine the contribution of TiO_2_ NPs vs. bulk counterpart in wastewater effluents, the estimated median range of Ti-containing NPs is 0.1–0.4 μg/L (0.5–2 μg/L maximum) in wastewater effluents based upon projected NP production of 2.5–10% (Westerhoff et al., 2011). Generally, TiO_2_ NPs present low classic toxicity with LC_50_ values in the high mg/L range or not achieved for a variety of species (Clemente et al., 2011). However, chemical concentrations that have no observable effects (NOEC) in terms of morphology are capable of disrupting sensitive endocrine signaling; a phenomenon which has been observed at very low NP concentrations (Colborn et al., 1993; Rajapakse et al., 2002; Hinther et al., 2010b). Chemicals or compounds that mimic or block the activity of naturally-circulating hormones with respect to transcriptional activation of their target genes are referred to as endocrine disrupting chemicals (EDCs) (Tabb and Blumberg, 2006). This can affect histone acetylation/deacetylation, DNA methylation, and transcriptional regulation, all of which may lead to developmental and reproductive anomalies (Tabb and Blumberg, 2006).

A process that lends itself well to the study of EDCs is amphibian metamorphosis, as it is driven solely by thyroid hormone (TH) (Shi, 2000; Hinther et al., 2010a). The TH pathway is highly conserved in vertebrates, so data on EDCs gathered from amphibian studies could be extended to other species, including humans. We have previously developed a rapid cultured tailfin (C-fin) assay (Hinther et al., 2010a) that is capable of evaluating TH signaling disruption. The C-fin assay involves collecting multiple biopsies from the tailfins of premetamorphic *Rana catesbeiana* tadpoles and culturing them *ex vivo* for 48 h in a concentration range of test chemical in the presence or absence of TH. A biopsy from each animal is cultured in each treatment condition, which allows the screening of multiple chemicals or concentrations of a certain chemical on the same individuals simultaneously without compromising complex tissue structure (Hinther et al., 2010a). Furthermore, inter-individual variation in response to the treatments can be determined because of the repeated-measures design of the assay.

Disruptions in TH signaling can be detected by examining changes in transcript levels of known TH-responsive genes. The TRs bind TH directly and are essential for execution of the cellular response to TH through gene activation or inactivation (Zhang and Lazar, 2000; Schreiber et al., 2001; Das et al., 2010). The genes encoding the TH receptors (TR) alpha (*thra*) and beta (*thrb*) are up-regulated upon TH exposure whereas the expression of *Rana* larval type I keratin (*rlk1*) decreases in the tail fin tissue of *R. catesbeiana* and *Rana clamitans* in response to TH (Domanski and Helbing, 2007; Hammond et al., 2013). Perturbations to the tail fin tissue in the absence of TH, such as induction of the cellular stress response, may also be interrogated through measurement of steady-state levels of mRNA from genes such as superoxide dismutase (*sod*) and catalase (*cat*), which are involved in the cellular metabolism of reactive oxygen species (ROS), and the general cell stress indicator, heat shock protein 30 (*hsp30*). The present study used the C-fin assay to evaluate the effects of three TiO_2_ NPs and micron-sized TiO_2_ particles (μ TiO_2_) on TH signaling and induction of cellular stress.

## Materials and methods

### Preparation of TiO_2_ NP, and μTiO_2_ test suspensions

The micro- and NPs are standard materials obtained through the Organization for Economic Cooperation and Development (OECD) Sponsorship program and the European Commission Joint Research Centre (JRC). These characterized stocks (see Table 1 for characteristics) were distributed to a variety of researchers from a common stock. The μ TiO_2_ test compound was prepared as a 1000 × stock of each treatment concentration, made by addition of powdered TiO_2_ (μTiO_2_; TIONA Titanium Dioxide Pigment, Cristal Global, Brussels, Belgium) to ddH_2_O and serially diluted to 10 μM, 1.0 μM and 0.1 μM based on the % TiO_2_ content listed in Table 1. The nano-sized TiO_2_ stock suspensions were made by addition of powdered M212 (UV-Titan M212 (NM-104), Sachtleben Chemie GmbH, Duisburg, Germany) or P25 (Aeroxide® TiO_2_ P25, Evonik Industries AG, Essen, Germany) TiO_2_ NP to ddH_2_O, or M262 (UV-Titan M262 (NM-103), Sachtleben) to DMSO. These preparations were then sonicated for 10 min using a Bioruptor UCD200 (Diagenode Inc., Sparta NJ, USA) on the “low” setting prior to 1000 × stock preparation. As above, the 1000 × stocks were made by serial dilution to 10 μM, 1.0 μM, and 0.1 μM TiO_2_, based on the % TiO_2_ content listed in Table 1. Therefore, the final nominal exposure concentrations were between 0.1 and 10 nM (equivalent to ~8–800 ng/L TiO_2_). These and the actual (see below) concentrations reflect a reasonable level of expected NP in the environment and permit direct comparison to previously published work on other NP types (Hinther et al., 2010b). For simplicity of presentation, the nominal concentrations are used throughout the present study.

**Table 1:** **Particle physical parameters and characterization**.

**Particle^a^**	**Nominal size (nm)^a^**	**TiO_2_ content (%)^a^**	**Surface coating^a^**	**Titanium concentration by ICP-MS (mg/L)^b^, ^c^**	**Aggregate diameter DLS (nm)^b^**		**Zeta potential (mV)^b^**	
					**dH_2_O**	**L15**	**dH_2_O**	**L15**
μTiO_2_(TIONA AT-1; anatase)	200–220	98.5	None	19.57 ± 1.37	235.0 ± 6.8	1285.2 ± 55.3	−16.46 ± 3.64	3.64 ± 3.62
P25 (80% anatase, 20% rutile)	21	99.5	None	23.47 ± 0.82	201.9 ± 3.1	1169.0 ± 64.7	8.15 ± 0.55	7.65 ± 0.30
M212 (rutile)	20	90	Alumina and glycerol (hydrophilic)	30.95 ± 0.96	470.8 ± 6.1	1270.5 ± 54.0	34.59 ± 1.17	7.92 ± 3.34
M262 (rutile)	20	89	Alumina and dimethicone (hydrophobic)	30.97 ± 1.21	268.3 ± 4.1	1095.0 ± 31.3	27.98 ± 1.59	1.8 ± 6.56

a Manufacturer specifications;

b triplicate measurements ± SD of stocks prepared at 80 mg/L (1 mM) TiO_2_;

c *nominal Ti concentration was 47.9 g/L (1 mM)*.

### Particle characterization

Stock solutions for characterization of each particle type were prepared with sonication (as above) at 1 mM (80 mg/L) in dH_2_O, based on manufacturer-declared TiO_2_ content. The M262 suspension included 0.1% (v/v) DMSO to facilitate solubilization. Average particle size, stability and Ti concentration were determined with dynamic light scattering (DLS), ζ-potential and inductively-coupled plasma mass spectrometry (ICP-MS), respectively. DLS and ζ-potential were measured using ZetaPALS (Brookhaven Instruments Corp., Holtsville, NY, USA) Particle Sizing Software and PALS Zeta Potential Analyzer, respectively. Stocks were sonicated and the average of three measurements at room temperature was taken for each particle type. For ICP-MS analysis, stocks were digested with nitric acid (20% m/m) and hydrogen peroxide (30% v/v) at 80°C for 4 h, followed by further sonication and incubation at 65°C for 3 h. Triton X100 was added to samples at 2% (v/v) to aid Ti detection of undigested particles (Shaw et al., 2013), performed with a Nexlon 300x (Perkin Elmer, Woodbridge, ON, Canada).

### Experimental animals

Taylor and Kollros (TK) (Taylor and Kollros, 1946) VI–VIII *R. catesbeiana* tadpoles were caught locally (Victoria, BC, Canada). The care and treatment of animals used in the present study was in accordance with the guidelines of the Canadian Council on Animal Care under the guidance of the Animal Care Committee of the University of Victoria. Animals were housed in the University of Victoria aquatics facility and maintained in 100 gallon fiberglass tanks containing recirculated water at 12°C. Tadpoles were fed daily with spirulina (Aquatic ELO-systems) and acclimated to lab conditions for 24 h prior to anaesthetization in a 0.1% (w/v) tricaine methanesulfonate (MS-222, Syndel Laboratories, Qualicum Beach, Canada).

### Organ culture of tailfin biopsies

The C-fin assay was performed as described previously (Hinther et al., 2010a). In brief, one assay per test chemical was run consisting of eight 6 mm biopsies from each of eight *R. catesbeiana* tadpoles. Therefore, eight exposure conditions could be tested with eight biological replicates per assay. Biopsies were cultured in 24 well Primaria culture plates (BD Biosciences, Mississauga, ON, Canada) in 1.0 mL of 70% strength Liebovitz's L15 medium (Life Technologies Inc., Burlington, ON, Canada) with 10 mM HEPES pH 7.5, 50 units/mL penicillin G sodium, 50 μg/mL streptomycin sulphate (Life Technologies) and 50 μg/mL neomycin (Sigma-Aldrich Canada Co., Oakville, ON, Canada) with particle vehicle control or one of three concentrations of a particular particle. The 1000 × TiO_2_ particle stocks were applied at 1 μ L/mL of media to final concentrations of 10, 1.0, or 0.1 nM (see Table 1 for additional information). The biopsies were incubated with the particles or their vehicle controls for 2 h prior to the addition of 10 nM T_3_ (Sigma-Aldrich Canada Co.) in 400 nM NaOH or 400 nM NaOH alone, final concentrations. After 48 h, the biopsies were harvested into 100 μ L RNAlater (Life Technologies) and stored at 4°C overnight before being moved to −20°C.

### Isolation of RNA and quantitation of gene expression

The biopsy tissues were loaded into 0.5 mL Safe-Lock Eppendorf tubes containing 300 μ L TRIzol reagent and a 1-mm diameter tungsten-carbide bead for mechanical disruption by a Retsch MM301 Mixer Mill (Thermo Fisher Scientific, Markham, ON, Canada) run at 20 Hz twice for 3 min, with the chambers rotated between cycles. Twenty microgram glycogen (Roche Diagnostics, Laval, QC, Canada) was added as a carrier prior to isopropanol precipitation to maximize RNA yield. Isolated RNA was resuspended in 10 μ L diethyl pyrocarbonate (DEPC)-treated (Sigma-Aldrich) RNase-free water and stored at −80°C. Complimentary DNA (cDNA) was synthesized using the High Capacity cDNA Reverse Transcription Kit (Life Technologies) from 1 μ g total RNA following the manufacturer's instructions. The reaction mixture was incubated at 25°C for 10 min, and then at 42°C for 2 h and 5 min at 85°C. The cDNA was diluted 20-fold with DEPC-dH_2_O and stored at −20°C.

**Figure 1:**
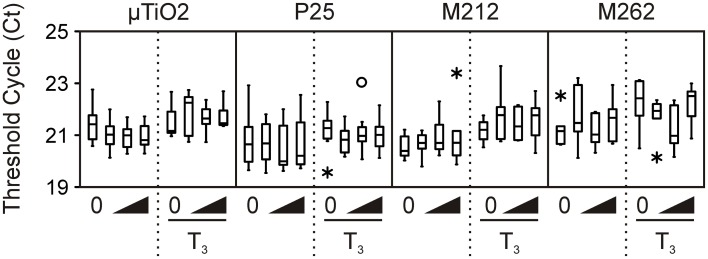
**Transcript levels of invariant normalizer gene *rpl8* in the μ TiO_2_ and TiO2 NP C-fin assays as indicated by the threshold cycle (Ct) values obtained by QPCR**. Bevels indicate increasing concentrations of the indicated compound in the absence or presence of T3. The medians are indicated by solid black lines within the boxes, where the box denotes the 25th and 75th percentiles, and the whiskers indicate the minimum and maximum values that lie within 1.5 box-lengths of the median. Outlier (between 1.5 and 3 box lengths) and extreme values (>3 box lengths) are shown as open circles or asterisks, respectively.

Transcript abundance was determined for TH-response genes [TH receptors *thra* and *thrb*, and *Rana* larval keratin 1 (*rlk1*)], stress-response genes [catalase (*cat*), heat shock protein 30 (*hsp30*), and superoxide dismutase (*sod*)], and the normalizer gene transcript [ribosomal protein L8 (*rpl8*)] using a MX3005P real-time QPCR system (Agilent Technologies Canada, Inc., Mississauga, ON, Canada). Primers and hydrolysis probes (Table 2) were ordered from Integrated DNA Technologies (IDT, Coralville, IA, USA). The *thra, thrb*, and *rpl8* QPCR reactions contained 0.01% Tween 20, 0.8% glycerol, 10 mM Tris-HCl (pH 8.3 at 20°C), 50 mM KCl, 3 mM MgCl_2_, 200 μ M dNTPs, 69.4 nM ROX reference dye (Invitrogen), one unit of Immolase DNA polymerase (Bioline USA Inc., Taunton, MA, USA), and 2 μ L of diluted cDNA (see Table 2 for sequences and amounts of primers and hydrolysis probes added). The *cat, hsp30*, and *sod* reactions were the same as the *thra*-containing reactions, but with the amount of primers and hydrolysis probes noted in Table 2. The *rlk1* QPCR reactions contained 0.01% Tween 20, 0.8% glycerol, 10 mM Tris-HCl (pH 8.3 at 20°C), 50 mM KCl, 3 mM MgCl_2_, 4,0000-fold dilution of SYBR Green I (Molecular Probes, Invitrogen), 200 μ M dNTPs, 69.4 nM ROX reference dye (Invitrogen), one unit of Immolase DNA polymerase (Bioline), 2 μ L of diluted cDNA (see Table 2 for amount of primers used). The thermocycle program for *thra, thrb, cat, hsp30,sod*, and *rpl8* consisted of an initial enzyme activation step at 95°C (9 min) followed by 40 cycles of 95°C denaturation (15 s), 64°C annealing (30 s), and 72°C elongation (30 s). The thermocycle program for *rlk1* was the same as above, except that the annealing temperature was 55°C (30 s) and the elongation phase was 45 s. A control lacking cDNA template was included to determine specificity of target cDNA amplification. Reactions were performed in quadruplicate for each sample and data averaged and normalized to expression of the invariant *rpl8* control transcript (Figure 1) using the comparative threshold method (ΔΔ Ct) method (Livak and Schmittgen, 2001). Amplification reaction integrity of the primers was confirmed by the presence of a single DNA product following gel electrophoresis and by digestion and restriction mapping of the product or through amplicon sequencing. Absence of genomic DNA (gDNA) contamination in the samples was confirmed through analysis of post-amplification denaturation of the *rlk1* reactions: amplicons generated from gDNA produce a denaturation profile different from that generated from cDNA template alone (data not shown). Use of the ΔΔ Ct method was validated by confirmation that the efficiencies of the target amplifications were approximately equal, i.e., that the slope of the line generated from the plotting of the log_2_ dilution vs. the Δ Ct values between the test gene transcript and *rpl8* is between −0.1 and 0.1 (Table 2).

**Table 2:** **Gene-specific DNA primers and hydrolysis probes for QPCR**.

**Gene target**	**GenBank accession #**	**Oligo name**	**Oligo sequence^a^**	**Amplicon size (bp)**	**pmol per reaction**	**ΔΔ Ct criteria**
*rpl8*	AY452063	AMM1	5′-AGGCAGGTCGTGCNTACCA-3′	89	1.5	107%^b^
		AMM2	5′-GGGATGTTCTACAGGATTCATAGC-3′		1.5	
		AMM3	5′-Cy5-AAACTGCTGGCCACGTGTCCGT-IABk-3′		1.5	
*thra*	L06064	AMM4	5′-TGATAAGGCCACAGGRTACCACTA-3′	141	4.5	0.037^c^
		AMM5	5′-CGGGTGATCTTGTCGATRA-3′		1.5	
		AMM6	5′-FAM-ACTATCCAGAAGAACCTGCACCCCTC-IABk-3′		4.5	
*thrb*	L27344	AMM7	5′-CTCATAGAAGAAAACAGAGAAAARAGA-3′	237	4.5	0.016^c^
		AMM8	5′-GAAGGCTTCTAAGTCCACTTTTCC-3′		1.5	
		AMM9	5′-HEX-CATGTGGCCACCAATGCACAGG-IABk-3′		4.5	
*hsp30*	U44894	AMM13	5′-GCCTCCACCAGACTTACCA-3′	238	4.5	0.057^c^
		AMM14	5′-TCTGTCTCCCTTTTCTTGTCG-3′		1.5	
		AMM15	5′-HEX-CCACCGCCCCTCAAGACAAATC-IABk-3′		4.5	
*cat*	GQ222411	AMM16	5′-GAATGGTTACGGCTCACACA-3′	176	1.5	0.056^c^
		AMM17	5′-TGGCAATGGCTTCATACAGAT-3′		1.5	
		AMM18	5′-Cy5-CAGGGCATCAGGAATCTGACGGT-IABk-3′		1.5	
*sod*	BT081775	AMM19	5′-CGAGCAGGAAGAAGATGGA-3′	323	4.5	0.078^c^
		AMM20	5′-CGCCTTTTCCCAAGTCATC-3′		1.5	
		AMM21	5′-ATTTCAACCCCCAAGGCAAGACC-3′		4.5	
*rlk1*	EF156435	DDKerF3	5′-GTTGGCGTTGGTGTTAGCGC-3′	336	5.0	0.080^c^
		DDKerRQ	5′-GGCACTGCTTCTTGCAACTTG-3′		5.0	

a Underlined sequence indicates the presence of an internal ZEN quencher;

b Amplification efficiency;

c *Slope of the ΔΔ Ct curve relative to rpl8 normalizer primers and hydrolysis probes were designed against the indicated sequences in GenBank*.

### Statistical analyses

The relative fold change data from the QPCR were determined to be not normally-distributed by the Shapiro–Wilk test, and to have unequal variances by Levene's test. Therefore, the non-parametric Friedman test for repeated measures was performed on all data sets using RStudio software (RStudio, Inc, Boston, MA, USA). Three comparisons were then made. The first was to assess the ability of the tissues from each animal to respond to T_3_ treatment. Adequate responsiveness of the tissues to T_3_ was determined by QPCR analysis: a 2-fold increase of *thrb* and 1.2-fold decrease of *rlk1* was the minimal accepted response. Animals that did not display this response (*n* = 5 in entire study) were removed from further analysis. The second comparison was to determine NP effects by pairwise analyses of each NP treatment alone relative to the vehicle control (NaOH). The third comparison was to identify NP-induced alteration of the normal T_3_ response by comparing T_3_ alone to each T_3_ + NP combination. All data were expressed as median values in box plots. Statistical significance was considered at *p*-value < 0.05.

## Results and discussion

For the past three decades, TiO_2_ was classified as an inert nuisance dust that was only a health risk if large amounts were inhaled (Ferin and Oberdorster, 1985). However, evidence that pulmonary exposure to TiO_2_ NPs in rats increased the incidence of lung tumors prompted the International Agency for Research on Cancer (IARC) to classify all TiO_2_ compounds as possibly carcinogenic to humans (class 2B carcinogens) (Baan et al., 2006). Other exposure studies on the effects of TiO_2_ NPs on biological systems have employed classic toxicological techniques, such as acute exposure of a model organism in order to calculate the LC_50_ or the use of immortalized cell lines(Jin et al., 2008; Zhu et al., 2008; Baun et al., 2009; Falck et al., 2009). The present study is the first to examine the effects of sublethal concentrations of TiO_2_ NPs and a micron-sized counterpart within the context and sensitive endpoints of amphibian tissues. Given that smaller particle sizes have been strongly associated with deleterious effects in biological systems (Griffitt et al., 2008; Jiang et al., 2008), the TiO_2_ NPs were expected to disrupt gene expression to a greater extent than the μ TiO_2_ particles.

The levels of titanium measured in the nominal 80 mg/L titanium dioxide particle stocks were 40.9–64.7% of the nominal value (Table 1). All of the particles studied were found to form aggregates when suspended in dH_2_O or L15 medium (Table 1). These aggregates were between 1095.0 and 1285.2 μm in diameter in L15, which was two to five-times greater than dH_2_O (Table 1). This aggregation is consistent with other studies of TiO_2_ NPs, which have found that these NPs rapidly coalesce in various natural and buffered liquid matrices (Keller et al., 2010; Thio et al., 2011). Interestingly, the aggregates formed by the NPs were generally comparable in size to those formed by the μ TiO_2_ (Table 1). The ζ-potential measurements indicated that the TiO_2_ particles were generally more stable in dH_2_O than L15 (Table 1). This decreased stability likely contributes to the large degree of aggregation noted by DLS analysis.

**Figure 2:**
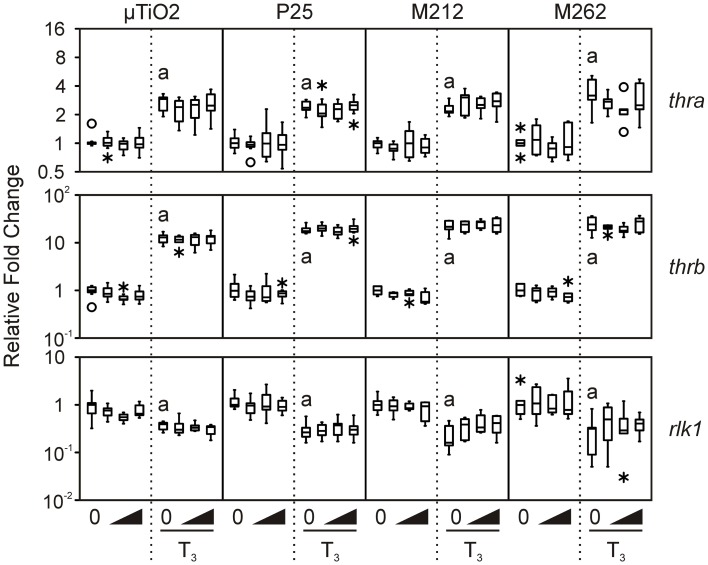
**Assessment of the effects of exposure to 0.1, 1.0, and 10 nM μ TiO_2_, P25, M212, or M262 on *thra, thrb*, and *rlk1* relative transcript levels in *R. catesbeiana* tailfin biopsies following 48 h exposure to NaOH vehicle or 10 nM T_3_**. Values represent fold-change of steady-state transcript levels relative to each individual's vehicle control baseline as measured by QPCR. Bevels indicate increasing concentrations of the indicated compoundin the absence or presence of T_3_. Significant response to T_3_ relative to the vehicle control is indicated by “a” when *p* ≤ 0.05. *thra*, thyroid hormone receptor α; *thrb*, thyroid hormone receptor β ; *rlk1*, *Rana* larval keratin type I. See Figure 1 legend for more graph details.

**Figure 3:**
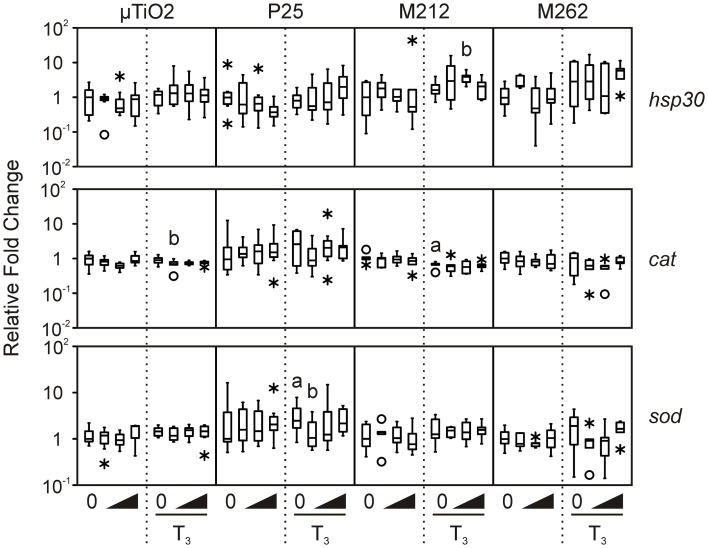
**Assessment of the effects of exposure to 0.1, 1.0, and 10 nM μ TiO_2_, P25, M212, or M262 on *hsp30, cat*, and *sod* relative transcript levels in *R. catesbeiana* tailfin biopsies following 48 h exposure to NaOH vehicle or 10 nM T_3_**. *cat*, catalase; *hsp30*, heat shock protein 30; *sod*, superoxide dismutase. See Figures 1, 2 legends for more graph details. Significance relative to the vehicle control for NPs alone or relative to the T_3_ only control for the T_3_-treated plus NP condition is indicated by “b”.

We then determined that the tissue biopsies were capable of responding to TH. As expected, treatment with 10 nM T_3_ consistently increased median levels of *thra* and *thrb* mRNAs by 1.7–6.7-fold and 8.2–48.5-fold, respectively, and decreased *rlk1* transcript levels by 1.2–20-fold across the four C-fin assays (Figure 2). In contrast, very little if any effect was observed for the three stress indicators, *hsp30*, *sod*, and *cat* (Figure 3). Hsp30 is involved in the cellular defense against heat shock and general stress in many eukaryotes (Nover et al., 1983; Krone and Heikkila, 1988; Helbing et al., 1996). During cellular stress *HSP30* and other small heat shock proteins form multimers and bind to denatured proteins to prevent their aggregation until the bound proteins are reactivated by other chaperones and allowed to refold (Heikkila, 2004). The level of *hsp30* mRNA was not significantly affected by T_3_ in any of the exposures. The cellular metabolism of ROS is essential to minimizing the destructive effects of molecules such as hydrogen peroxide and superoxide within the cell. ROS are a byproduct of energy metabolism under normal conditions, but ROS levels can increase dramatically during environmental stress and cause damage to the cellular structure and contents (Valavanidis et al., 2006). The dismutation of superoxide to hydrogen peroxide and molecular oxygen is catalyzed by *sod*, while *cat* catalyzes the decomposition of hydrogen peroxide to water and oxygen. The complementary activity of these latter two enzymes means that they are typically coregulated by ROS, both at the mRNA and protein level (Mates et al., 1999; Rodriguez et al., 2004). Induction of stress-response genes was minimal in response to T_3_ treatment: steady-state levels of *sod* transcripts were increased modestly by T_3_ in the P25 exposure set (1.9-fold, Figure 3), and *cat* transcript levels were slightly decreased in the M212 exposure set (1.5-fold, Figure 3).

Exposure to μ TiO_2_, P25, M212, or M262 did not affect the relative abundance of any of the TH-responsive mRNAs examined when tested alone or together with an application of 10 nM T_3_ (Figure 2). These results are consistent with those observed previously for nanozinc oxide (Hinther et al., 2010b), but are in contrast with previously observed perturbations due to nanosilver and cadmium telluride quantum dots (QDs) (Hinther et al., 2010b).

In the vast majority of TiO_2_ NP and μ TiO_2_ exposures, no effect on the stress markers was observed (Figure 3), except in the presence of T_3_ with either μ TiO_2_ (0.1 nM: 1.3-fold decrease in *cat*), P25 (0.1 nM: 2.4-fold decrease in *sod*), or M212 (1.0 nM: 2.1-fold increase in *hsp30*) (Figure 3). The minor transcriptional perturbations varied between particles with no dose response or substantial fold changes (Figures 2, 3). Although the unique chemical properties of each could contribute to differential responses between NP types, no overall pattern was observed.

The two most common crystal structures of TiO_2_ (rutile and anatase) have different photocatalytic and toxic qualities of which the anatase form is considered to be the more potent of the two (Nakagawa et al., 1997; Sayes et al., 2006). The P25 NPs are predominantly anatase, whereas the M212 NPs are solely rutile (Table 1). Both particles had limited effects on expression of the stress-response genes examined (Figure 3), so neither crystal structure would appear to be especially potent in the present context.

The principal difference between the M212 and M262 NPs is their surface functionalizations which give them contrasting interactions with water (Table 1). The surface interactions of particles with their media and each other have been associated with differential effects in chemical and toxicological assays (Tiano et al., 2010). While an increase in *hsp30* mRNA detected in the M212 exposure would suggest that the hydrophilic coating may be slightly more stressful to the biopsies in this assay, this difference is minor and agrees with the conclusion of Bolis et al. that the hydrophilic nature of the particle would not appear to be a good predictor of the disruptive potential of TiO_2_ NPs (Bolis et al., 2012). Moreover, all of the particles tested aggregated to similar degrees in the L15 medium suggesting that the surface area-dependent increase in reactivity associated with NPs was not a contributing factor.

A recent study on nanozinc oxide, nanosilver, and QDs, showed a substantial range between NP types in altering stress-induced transcripts in cultured *R. catesbeiana* tailfin biopsies (Hinther et al., 2010b). The nanozinc oxide had no effect, whereas the QDs caused massive increase of *hsp30* transcripts and depressed *cat* transcript levels (Hinther et al., 2010b). The data from the present study indicate TiO_2_ NPs behave more like nanozinc oxide and the minor effects observed do not appear to depend on the surface functionalization or crystal structure.

It has been proposed that ROS generation is the primary cause of cellular perturbation and damage by TiO_2_ NPs (Gurr et al., 2005; Hao et al., 2009). P25 NPs have been shown to produce ROS *in vitro*, although at 1 μ g/L ROS levels were not significantly increased over the control (Du et al., 2011). ROS production by TiO_2_ NPs is enhanced under UV light, and in comparative studies, absence of excitation by UV radiation resulted in a lower degree of cytotoxicity and ROS production (Cermenati et al., 1997; Afaq et al., 1998; Dick et al., 2003; Tiano et al., 2010; Zhang et al., 2012). The C-fin assay is stored in a dark incubator during the 48 h exposure, so the levels of UV-generated ROS are negligible as is shown by the lack of stress response. Future studies should incorporate light exposure and simulate NP aging, both of which could alter the particle's biological effects on amphibian tissues. However, the present study indicates that in the absence of UV exposure, environmentally-relevant concentrations of TiO_2_ NPs are relatively inert.

### Conflict of interest statement

The authors declare that the research was conducted in the absence of any commercial or financial relationships that could be construed as a potential conflict of interest.
